# Motherese in Interaction: At the Cross-Road of Emotion and Cognition? (A Systematic Review)

**DOI:** 10.1371/journal.pone.0078103

**Published:** 2013-10-18

**Authors:** Catherine Saint-Georges, Mohamed Chetouani, Raquel Cassel, Fabio Apicella, Ammar Mahdhaoui, Filippo Muratori, Marie-Christine Laznik, David Cohen

**Affiliations:** 1 Department of Child and Adolescent Psychiatry, Pitié-Salpêtrière Hospital, Université Pierre et Marie Curie, Paris, France; 2 Institut des Systèmes Intelligents et de Robotique, Centre National de la Recherche Scientifique 7222, Université Pierre et Marie Curie, Paris, France; 3 Laboratoire de Psychopathologie et Processus de Santé (LPPS, EA 4057), Institut de Psychologie de l'Université Paris Descartes, Paris, France; 4 IRCCS Scientific Institute Stella Maris, University of Pisa, Pisa, Italy; 5 Department of Child and Adolescent Psychiatry, Association Santé Mentale du 13ème, Centre Alfred Binet, Paris, France; Birkbeck, University of London, United Kingdom

## Abstract

Various aspects of motherese also known as infant-directed speech (IDS) have been studied for many years. As it is a widespread phenomenon, it is suspected to play some important roles in infant development. Therefore, our purpose was to provide an update of the evidence accumulated by reviewing all of the empirical or experimental studies that have been published since 1966 on IDS driving factors and impacts. Two databases were screened and 144 relevant studies were retained. General linguistic and prosodic characteristics of IDS were found in a variety of languages, and IDS was not restricted to mothers. IDS varied with factors associated with the caregiver (e.g., cultural, psychological and physiological) and the infant (e.g., reactivity and interactive feedback). IDS promoted infants’ affect, attention and language learning. Cognitive aspects of IDS have been widely studied whereas affective ones still need to be developed. However, during interactions, the following two observations were notable: (1) IDS prosody reflects emotional charges and meets infants’ preferences, and (2) mother-infant contingency and synchrony are crucial for IDS production and prolongation. Thus, IDS is part of an interactive loop that may play an important role in infants’ cognitive and social development.

## Introduction

Motherese, also known as infant-directed speech (IDS) or “baby-talk”, refers to the spontaneous way in which mothers, fathers, and caregivers speak with infants and young children. In a review of the various terms used to denote young children’s language environments, Saxton suggested the preferential use of “infant- or child-directed speech” [1]. In 1964, a linguist [2] defined “baby-talk” as “a linguistic subsystem regarded by a speech community as being primarily appropriate for talking to young children”. He reported that “baby talk” was a well-known, special form of speech that occurred in a number of languages and included the following 3 characteristics: (1) intonational and paralinguistic phenomena (e.g., a higher overall pitch), (2) words and constructions derived from the normal language (e.g., the use of third person constructions to replace first and second person constructions), and (3) a set of lexical items that are specific for baby talk. He provided a precise, documented study of IDS across several different languages. Since then, infant-directed speech has been studied extensively across a number of interactive situations and contexts, especially by researchers interested in understanding language acquisition. A recent review of “baby-talk” literature focused on phonological, lexical and syntactic aspects of the input provided to infants from the perspective of language acquisition and comprehension [3]. Although Snow, in a review of the early literature on motherese [4], claimed that “language acquisition is the result of a process of *interaction* between mother and child, which begins early in infancy, to which the child makes as important a contribution as the mother, and which is crucial to cognitive and *emotional* development as well as language acquisition”, few experimental findings have sustained this assertion. Recent progresses in cognitive science and in interactional perspective suggest, however, that infant cognitive development is linked with social interaction (e.g., Kuhl et al., 2003). Motherese could be a crossroad for such a linkage. Here, we aim to review the available evidence relevant to motherese from an interactional perspective, with a specific focus on children younger than 2 years of age. In contrast with Soderstrom’s review (2007), we focus more preferentially on motherese’s prosodic and affective aspects to determine the factors, including interactive ones, associated with its production and variations, its known effects on infants and its suspected functions aside from language acquisition.

## Methods

**Figure 1:**
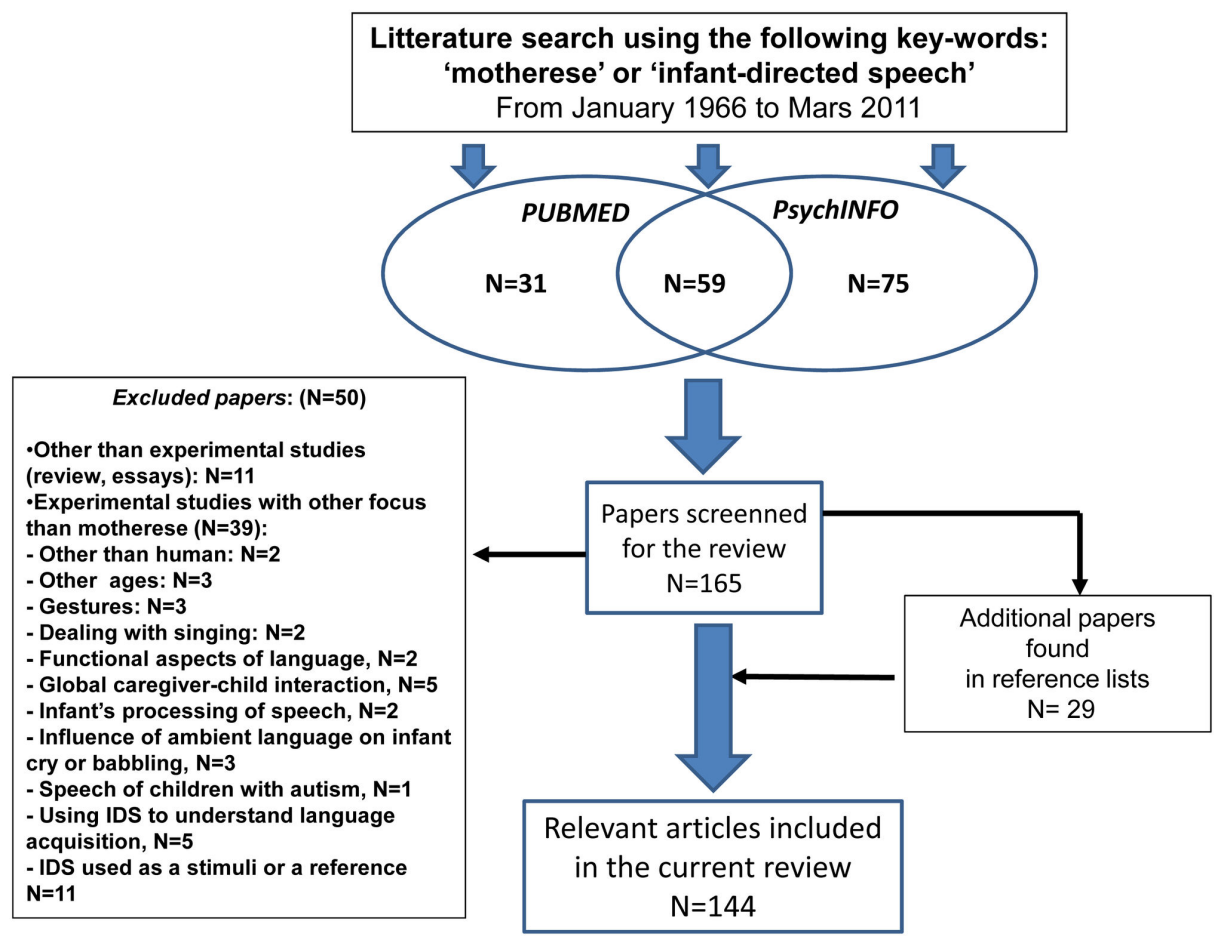
Diagram flow of the literature search.

We searched the PubMed and PsycInfo databases from January 1966 to March 2011 using the following criteria: journal article or book chapter with ‘‘motherese’’ or ‘‘infant-directed speech’’ within the title or abstract, published in the English language and limited to human subjects. A diagram summarizing the literature search process is provided in Figure 1. We found 90 papers with PubMed and 134 with PsycInfo, of which 59 were shared across the databases, for a total of 165 papers. We excluded 50 papers because 11 were reviews or essays and 39 were experimental studies that did not aim to improve knowledge on IDS as they addressed other aims (see details in Annex S1). We found an additional 29 references by screening the reference lists of the 115 papers, leading to a total of 144 relevant papers.

## Results

### 1: General comments

Table 1 lists the relevant studies and the number of subjects included according to each domain of interest. The following observations are evident: (1) certain points are well documented (e.g., IDS’s effect on language acquisition), whereas others have received less support (e.g., IDS production according to gender and the course of infants’ preference for IDS); (2) the sample sizes between studies range from 1 to 276 with 1/3 of the studies having N≤15, 1/3 having 15<N<40, and 1/3 having N≥40; and (3) methodologies vary greatly between studies with regard to design and sample characteristics (e.g., the type of locator and infants’ ages). The results are presented in several sections. Concerning IDS production, we will first review its general characteristics and, then, its variations according to maternal language, infants’ age, gender, vocalizations, abilities and reactivities, and parental individual differences. For IDS’s effects on infants, we listed the following 4 main functions of IDS: communicating affect, facilitating social interaction through infants’ preferences, engaging and maintaining infants’ attention, and facilitating language acquisition. The discussion incorporates selected articles dealing with theoretical considerations and those that included the boundaries of the concept of motherese.

**Table 1:** Characteristics of the studies included in the review.

**Author, year**		N* subjects			**Design of the study**	Main objective to explore or assess… (**motherese features and variations**)
Durkin 1982		18		Cross-sectional observational		Functions of use of proper names
Fernald 1984		24		Paired comparisons IDS/simulated IDS/ADS		Prosodic features according to infant feed-back
Fisher 1995		20		Paired comparisons IDS/ADS		Prosodic features on new/given words
Soderstrom 2008		2		Longitudinal case series		Prosodic and linguistic features
Fernald 1991		18		Paired comparisons IDS/ADS		Prosodic features on focused words
Ogle 1993		8		Cross- overIDS/ADS (electrolaryngography)		Prosodic features (F0 measures)
Fernald 1989a		30		Paired comparisons IDS/ADS		Prosodic features for mothers and fathers across 6 languages
Niwano 2003b		3		Paired comparisons IDS/ADS		Prosodic features for mothers and fathers, + infant’s responses
Shute 1999		16		Paired comparisons IDS/ADS		Prosodic features for fathers speaking and reading aloud
Shute 2001		16		Paired comparisons IDS/ADS		Prosodic features for grandmothers speaking and reading aloud
Nwokah 1987		16		Case-control (Mother/maid)		Linguistic and functional features of maids’ IDS
Katz 1996		49		Paired comparisons with pragmatic categories of IDS		Prosodic contours according to intention
Stern 1982		6		Case-series		Prosodic contours according to intention, grammar, and context
Papoušek 1991		20		Case-control (Chinese/English)		Prosodic contours according to context in different languages
Slaney 2003		12		Paired comparisons (IDS with various intentions)		Acoustic measures according to affect (automatic classification)
Trainor, 2000		96		Paired comparisons IDS/ADS with various emotions		Links between IDS and affective expression
Inoue, 2011		24		Paired comparisons IDS/ADS		Wether Mel-frequency cepstral coefficients discriminate IDS from ADS
Mahdhaoui 2011		11		Paired comparisons IDS/ADS		Automatic detection based on prosodic and segmental features
Cristia, 2010		55		Paired comparisons IDS/ADS		Enhancement of consonantal categories
Albin 1996		16		Paired comparisons IDS/ADS		Lengthening of word-final syllables
Swanson 1992		15		Paired comparisons IDS/ADS		Vowel duration of content words as opposed to function words
Swanson 1994		22		Paired comparisons IDS/ADS		Vowel duration of function-word in utterance final position
Englund 2006		6		Longitudinal Paired comparisons IDS/ADS		Vowels and consonant specification throughout the first semester
Englund, 2005a		6		Longitudinal Paired comparisons IDS/ADS		Spectral attributes and duration of vowels throughout a semester
Englund, 2005b		6		Longitudinal Paired comparisons IDS/ADS		Evolution of voice onset time in stops throughout a semester
Lee 2010		10		Case-control (IDS/ADS)		Segmental distribution patterns in English IDS
Shute 1989		8		Paired comparisons IDS/ADS		Pitch Variations in British IDS (compared to American IDS)
Segal 2009		11		Longitudinal descriptive study		Prosodic and lexical features in Hebrew IDS
Lee 2008		10		Paired comparisons IDS/ADS		Segmental distribution patterns in Korean IDS
Grieser 1988		8		Paired comparisons IDS/ADS		Prosodic features in a tone language IDS (Mandarin Chinese)
Liu 2007		16		Paired comparisons IDS/ADS		Exaggeration of lexical tones in Mandarin IDS
Fais 2010		10		Paired comparisons IDS/ADS		Vowel devoicing in Japanese IDS
Masataka 1992		8		Paired comparisons IDS/ADS		Rhythm, repetition and gestual exaggeration in Japanese sign language
Reilly 1996		15		Longitudinal descriptive study		Competition between affect and grammar in American sign language
Werker 2007		30		Cross-language comparison		Differences in distributional properties of vowel phonetic categories
Kitamura 2003		12		Longitudinal Paired comparisons IDS/ADS		Pitch and communicative intent according to age
Stern 1983		6		Longitudinal case-series		Prosodic features evolution
Niwano 2002b		50		Longitudinal case-series		Pitch and Prosodic contours according to age
Liu 2009		17		Longitudinal Paired comparisons IDS/ADS		Prosodic and phonetic features according to age
Kajikawa 2004		2		Longitudinal case-series		Adult conversational style (Speech overlap) emergence in Japanese IDS
Amano 2006		5		Longitudinal case-series		Changes in F0 according to infant age and language acquisition stage
Snow 1972		12/24/6		Paired comparisons IDS/CDS		Linguistic features according to children age
Kitamura 2002		22		Longitudinal Paired comparisons IDS/ADS		Pitch according to infant age and gender in English and Thaï languages
Braarud 2008		32		Paired comparisons synchrony/dyssynchrony		IDS quantity according to infant feed-back and synchrony
Smith 2008		18		Controlled trial (2 experimental groups)		Pitch variations according to infant feed-back from the pitch
Shimura 1992		8		Correlation study		Between mother and infant vocalizations (pitch, duration, latency, melody)
Van Puyvelde 2010		15		Correlation study		Between mother and infant vocalizations (pitch, melody)
McRoberts 1997		1		Longitudinal case-study		Mother, father and infant adjustment of pitch vocalizations during interaction
Reissland 1999		13		Case-control (premature/term infants)		Timing and reciprocal vocal responsiveness of mothers and infants
Niwano 2003a		1		Paired comparisons (mother with twins)		Pitch and contours variations according to infant reactivity
Reissland 2002		48		Case-control (age) + Correlation study		Pitch of IDS surprise exclamation according to infant age/reaction to surprise
Lederberg 1984		15		Paired comparisons deaf/hearing children		Adult adjustment in interaction with deaf children
Fidler, 2003		36		Case-control (Down syndrome/other MR)		Pitch’s mean and variance in parental IDS to Down syndrome/other MR
Gogate 2000		24		Case-control (5-8;9-17;21-30 months)		Multimodal IDS according to infants' levels of lexical-mapping development
Kavanaugh 1982		4		Longitudinal case-series		Mother/father linguistic input according to apparition of productive language
Bohannon 1977		20		Correlation study		MLU of IDS according to child’s feed-back of comprehension
		20		Paired comparisons (manipulating feed-back)		
Bergeson 2006		27		Case-control (cochlear implant/control)		IDS adjustment (pitch, MLU, rhythm) according to childs' hearing experience
Kondaurova 2010		27		Longitudinal case-control		IDS adjustment according to child’s hearing experience and age
Ikeda 1999		61		Paired comparisons IDS/ADS		Variations according to various life experience (especially having sibling)
Hoff 2005		63		Prospective study		Variations of linguistic input and teaching practices according to parental socio-
		662		Cross-sectional study		- economic status or education, and repercussions on child vocabulary
Hoff-Ginsberg 1991		63		Cross-sectional study		Variations of input according to parental socio-economic status (SES)
Matsuda 2011		65		Correlation study		Functional RMI of adults listening to IDS according to gender, parental status
Gordon 2010		160		Prospective study		Oxytocin level according to infant’s age and correlation with parenting
Bettes 1998		36		Case-control		Maternal behavior (including IDS prosody) according to depression status
Herrera 2004		72		Case-control		IDS content and touching according to maternal depression status
Kaplan 2001		44		Correlation study		Variations according to maternal age and depression status
Wan 2008		50		Case-control		Variations of IDS characteristics according to maternal schizophrenia status
Nwokah 1999		13		Case-control		IDS amount, structure, and content in maids compared with mothers
Burnham 2002		12		Paired comparisons IDS/ADS/petDS		Pitch, affect (intonation + rhythm) and hyperarticulation in IDS versus petDS
Green 2010		25		Paired comparisons IDS/ADS		Lip movements
Rice 1986		2		Case-series		Description of speech in educational television programs compared with CDS
**Author, year**	N* subjects		**Design of the study**			Main objective to explore or assess**… motherese effects**
Fernald 1989b	5		Paired comparisons IDS/ADS with various intentions			Adult’s detection of communicative intent according to prosodic contours
Bryant 2007	8		Paired comparisons IDS/ADS with various intentions			Adult’s detection of communicative intent according to prosodic contours
Fernald 1993	120		Paired comparisons IDS/ADS with various intentions			Communication of affect ( to infants) through prosodic contours
Papousek 1990	32		Paired comparisons approval/disapproval intent			Communicating affect (looking response) through prosodic contours
Santesso 2007	39		Paired comparisons with various affects			Psycho-physiological (ECG, EEG) responses to IDS with various affects
Monnot 1999	52		Correlation study			IDS effects on infant’s development level and growth parameters
Santarcangelo 1988	6/4		Correlation study + paired comparisons IDS/ADS			Developmentally disabled children’s preference (responsiveness, eye-gaze)
Werker 1989	60		Paired comparisons IDS/ADS with males/females			Infant’s preference (looking, facial expression) for male and female IDS
Schachner 2010	20		Paired comparisons IDS/ADS			Subsequent visual infant’s preference for the speaker
Masataka 1998	45		Paired comparisons IDS/ADS			Infant’s preference for infant-directed (versus adult-directed) Sign Language
Cooper 1993	96		Paired comparisons IDS/ADS			1 month-old infant’s preference for IDS
Cooper 1990	28		Paired comparisons IDS/ADS			Experimental (looking producing IDS) testing of 0-1 month-olds’ preference
Pegg 1992	92		Paired comparisons IDS/ADS			Young infant’s attentional and affective preference for male and female IDS
Niwano 2002a	40		Paired comparisons with manipulated IDS			Infant’s preference (through eliciting vocal response)
Hayashi 2001	8		Longitudinal paired comparisons IDS/ADS			Developmental change in infant’s preference (according to age)
Newman 2006	90		Paired comparisons IDS/ADS at 3 ages/2 noise levels			Change in infant’s preference according to developmental age and to noise
Panneton 2006	48		Paired comparisons with manipulated IDS at 2 ages			Change in determinants of infant’s preference according to developmental age
Cooper 1997	20/20/23		3 Paired comparisons IDS/ADS in various conditions			Change in infant’s preference according to age and speaker (mother/stranger)
Hepper 1993	30		Paired comparisons IDS/ADS			New-born’s preference for maternal IDS or ADS
Kitamura 2009	24		3 Paired comparisons IDS with various contours			Change in determinants of infant’s preference according to developmental age
Kaplan 1994	45/80		2 Paired comparisons IDS with various contours			Change in determinants of infant’s preference according to developmental age
Spence 2003	42		3 Paired comparisons IDS with various intents			Intent categorization ability according to age (4 months/6 months)
Johnson 2002	210		Paired comparisons IDS/ADS (prosody or content)			Adult’s preference for IDS/ADS according to history of head injury
Cooper 1994	12/20/20/16		4 Paired comparisons manipulated IDS/ADS			Do pitch contours determine 1-month-olds’ preference for IDS?
Fernald 1987	20		Paired comparisons with manipulated IDS			Do pitch, amplitude or rhythm determine 4-month-olds’ preference for IDS?
Leibold 2007	57		Paired comparisons with manipulated sounds			Acoustic determinants of 4-month-olds’ preference for IDS
Trainor 1998	16		Paired comparisons low or high pitched songs			Acoustic determinants of infant’s preference for IDS
Singh 2002	36		Paired comparisons IDS/ADS with various affects			Does affect (emotional intensity) determine infant’s preference for IDS ?
McRoberts 2009	144/62/24/48		4 Paired comparisons with manipulated IDS /ADS			Does repetition influence infant’s preference for age-inappropriate IDS/ADS?
Saito 2007	20		Paired comparisons IDS/ADS			Does IDS activate brain of neonates (near-infra-red spectroscopy)?
Kaplan 1996	104/78/80		3 Paired comparisons IDS/ADS			Does IDS (paired with what facial expressions) increase conditioned learning?
Kaplan 1995	77/26		Paired comparisons IDS/ADS			Does IDS engage and maintain infant’s attention?
Senju 2008	20		Paired comparisons IDS/ADS			Does IDS engage infant’s joint attention (eye-tracking) ?
Nakata 2004	43		Paired comparisons maternal IDS/maternal singing			Does IDS engage and maintain infant’s attention over singing?
Kaplan 2002	12		Paired comparisons depressed/non depressed IDS			Does IDS increase conditioned learning, according to mother depression?
Kaplan 1999	225		Controlled trials with IDS varying in quality			Does IDS increase conditioned learning, according to mother depressiveness?
Kaplan 2010a	134		Case-control			Does mother depression duration affect infant’s learning with normal IDS?
Kaplan 2004	40		Paired comparisons with maternal/female/male IDS			Does IDS speaker’s gender affect learning by infants of depressed mothers?
Kaplan 2010b	141		Case-control (2x2 ANOVA)			How marital status and mother depression affect learning with male IDS?
Kaplan 2007	39		Case-control			Does father depression affect infant’s conditioned learning with paternal IDS?
Kaplan 2009	55		Correlation study			Does maternal sensitivity affect infant’s learning with maternal IDS?
Karzon 1985	192		Controlled trials: IDS/manipulated IDS/ADS			Do supra-segmental features of IDS help polysyllabic discrimination?
Karzon 1989	64		Controlled trials: falling/rising contours			Does IDS prosody help syllabic discrimination and how?
Vallabha 2007	-		Automatic computed vowels categorization			Does IDS prosody help categorization of sounds from the native language?
Trainor 2002	96		Controlled trials			How IDS high pitch/ IDS exaggerated contours help vowel discrimination?
Hirsh-Pasek 1987	16/24		Paired comparisons with manipulated IDS			Does IDS prosody help to segment speech into clausal units?
Kemler Nelson 1989	32		Randomized controlled trials with IDS/ADS			Does IDS/ADS prosody help to segment speech into clausal units?
Thiessen 2005	40		Controlled trials with IDS/ADS			Does IDS prosody help word segmentation?
D’Odorico 2006	18		Case-control late-talker/typical peers			Does (prosodic and linguistic) maternal input help language acquisition?
Curtin 2005	24		Serie of 5 experiments			Does lexical stress help language acquisition (speech segmentation)?
Singh 2008	40		Serie of 4 experiments (controlled trials)			Does IDS vocal affect help word recognition?
Colombo 1995	27		Paired comparisons with manipulated sounds			Does F0 modulation in IDS help words recognition in a noisy ambient?
Zangl 2007	19/17		Paired comparisons IDS/ADS at 2 ages			Does IDS/ADS prosody activate brain for familiar and unfamiliar words?
Song 2010	48		Paired comparisons IDS/manipulated IDS			Does IDS rhythm/hyper-articulation/pitch amplitude help word recognition?
Bard 1983	94		4 Paired comparisons IDS/ADS with adult listeners			Does IDS help word recognition, according to word contextual predictability?
Bortfeld 2010	16/32/24/80		4 paired comparisons IDS words with various stress			Does emphatic stress in IDS prosody help word recognition ?
Kirchhoff 2005	Automatic		Paired comparisons IDS/ADS words			Does IDS prosody help automatic speech recognition ?
Singh 2009	32		Longitudinal paired comparisons (?) IDS/ADS			Does IDS prosody help word recognition over the long-term?
Golinkoff 1995	61/79		Randomized controlled trials IDS/ADS			Does IDS prosody help adult word recognition in an unfamiliar language?
Newport 1977	12		Longitudinal prospective correlation study			Does maternal IDS linguistic properties predict child language acquisition?
Gleitman 1984	6/6		Same as Newport 1977			New analyses on the same data but with 2 age-equated groups
Scarborough 1986	9		Longitudinal prospective correlation study			Does maternal IDS linguistic properties predict child language acquisition ?
Furrow 1979	7		Longitudinal prospective correlation study			Does maternal IDS linguistic properties predict child language acquisition ?
Rowe 2008	47		Prospective study			Does input according to parental SES affect child’s vocabulary?
Hampson 1993	45		Longitudinal prospective study			Does maternal IDS linguistic properties predict language acquisition ?
Waterfall 2010	12		Longitudinal study + computational analysis			Does IDS linguistic properties help language acquisition?
Onnis 2008	44/29		Randomized controlled trials Overlap/not			Does IDS properties (overlapping sentences) help word/grammar acquisition?
Fernald 2006	24		Paired comparisons with words isolated/not			Which properties (isolated words/short sentences) help language acquisition?
Kempe 2005	72/168		Randomized controlled trials Invariance/not			Does IDS diminutives (final syllable invariance) help word segmentation?
Kempe 2007	486		Randomized controlled trials Invariance/not			Does IDS diminutives (final syllable invariance) help word segmentation?
Kempe 2003	46		Paired comparisons with diminutives/not			Does IDS diminutives help gender categorization?
Seva 2007	24/22		Paired comparisons with diminutives/not			Does IDS diminutives help gender categorization?

* *N refers* to number of adults speaking in motherese production studies, and to infants (or sometimes adults) listening in motherese effects studies.

IDS=Infant-Directed Speech: ADS=Adult-Directed Speech

### 2: Motherese characteristics

The general linguistic and paralinguistic characteristics of motherese have been described in several previous works. Compared with Adult Directed Speech (ADS), IDS is characterized by shorter [5-7], linguistically simpler, redundant utterances, which include isolated words and phrases, a large number of questions [7], and the frequent use of proper names [8]. Regarding rhythm and prosody, longer pauses, a slower tempo, more prosodic repetitions, and a higher mean f0 (fundamental frequency: pitch) and wider f0-range have been reported [5,6,9], with these findings supported by electro-laryngographic measures [10]. Similar patterns of IDS have been observed for fathers and mothers across various languages [11-13], except with regard to the wider f0-range, and also for grandmothers interacting with their grandchildren [14]. In contrast, a maid’s IDS differs significantly from a mother’s IDS with regard to the amount and types of utterances present [15].

Prosodic contours vary according to mothers’ intentions. Adults hearing content-filtered speech [16] or a language that they do not speak [17] were able to use the intonation to identify a mother's intent (e.g., attention bid, approval, and comfort) with higher accuracy in IDS than in ADS. The prosodic patterns of IDS are more informative than those of ADS, and they provide infants with reliable cues about a speaker’s communicative intent. Indeed, f0 contour shape and f0 summary features (i.e., mean, standard deviation, and duration) discriminate the pragmatic categories (e.g., attention, approval, and comfort) from each other [18]. Mothers of 2- to 6-month-old infants use rising contours when seeking to initiate attention and eye contact, but they use sinusoidal and bell-shaped contours when seeking to maintain eye contact and positive affect with an infant who is already gazing and smiling. They also use specific contours for different sentence types, such as rise contours for yes-no questions, fall contours for "wh" questions and commands, and sinusoidal-bell contours for declarative sentences [19]. Moreover, across different languages, the same types of contours convey the same types of meanings, which include arousing/soothing, turn-opening/turn-closing, approving/disapproving, and didactic modeling [20]. Using pitch and spectral-shape measures, a Gaussian mixture-model discriminator designed to track affect in speech classified ADS (neutral affect) and IDS with more than 80% accuracy and further classified the affective message of IDS with 70% accuracy [21]. Indeed, the prosodic features of IDS are related to the widespread expression of emotion towards infants compared with the more inhibited expression of emotion evident in typical adult interactions. Few acoustic differences exist between IDS and ADS when expressing love, comfort, fear, and surprise, yet robust differences exist across these emotions [22]. Furthermore, in contrast with ADS, speech and laughter often co-occur in IDS [23]. Finally, IDS directed at 6-month-old human infants and pet-directed speech (PDS) [24] are similar in terms of heightened pitch and greater affect (i.e., intonation and rhythm). However, only IDS contains hyper-articulated vowels, which most likely aids in the emergence of language in human infants with both pragmatic and language teaching functions. Thus, IDS prosody appears to be crucial for communicating parents’ affect and intentions in a non-verbal way. 

In motherese, prosodic and phonetic cues highlight syntax and lexical units, and prosody provides cues regarding grammatical units at utterance boundaries and even at utterance-internal clause boundaries [7]. Indeed, mothers reading to their children lengthen vowels for content words [25] and function words when they appear in a final position [26]. Mothers also position target words on exaggerated pitch peaks in the utterance-final position [27] but lengthen final syllables, even in utterance-internal positions [28], 

Although IDS analyses generally focus on supra-segmental prosodic cues, recent works aiming to computerize the recognition of motherese show that IDS’s segmental and prosodic characteristics are intertwined [29,30]. The vocalic and consonantal categories are enhanced even when controlling for typical IDS prosodic characteristics [31]. Throughout the first 6 months, the vowel space is smaller and the vowel duration is longer, with some consonants also differing in duration and voice onset time. These characteristics may enhance both auditory and visual aspects of speech [32-34]. Along with acoustic characteristics, visual cues seem to be a part of motherese, which suggests that hyper-articulation in natural IDS may visually and acoustically enhance speech. Indeed, lip movements are larger during IDS than ADS [35]. 

### 3: Variations in motherese characteristics

**3.1. According to language**

Specific forms of IDS are evident across various languages, including Western European languages [11,36,37], Hebrew [38], Korean [39], Mandarin [40,41], Japanese [42] and even American Sign Language (ASL) between deaf mothers and their deaf children [43-45]. Although general trends in the form of IDS exist, they may be mediated by linguistic and cultural factors. French, Italian, German, Japanese, British English and American English IDS share some general features (i.e., higher mean f0, greater f0 variability, shorter utterances, and longer pauses) but maintain distinct characteristics. For example, American IDS exhibits the most extreme prosodic modifications [11], whereas British IDS exhibits smaller increases in vocal pitch [37] and has language-specific segmental distribution patterns when compared with Korean IDS [36]. Moreover, observations suggest that mothers adapt their IDS to the language-specific needs of their infants, for example, Japanese mothers alter phonetic cues that are more relevant in Japanese, whereas English mothers alter cues that are more relevant in English [46]. However, when a conflict arises between motherese features and language specificities (because some IDS features may disturb language salience), IDS tends to preserve the cues that are essential in ADS. Indeed, IDS prosody does not distort the acoustic cues essential to word meaning at the syllable level in Mandarin, which is a “tone language” [41], and this is also evident for the Japanese vowel devoicing [42]. When there is a conflict between grammatical and affective facial expressions in ASL IDS, mothers shift from stressing affect to grammar around the time of their children's second birthday [45]. 

**3.2. According to infants’ age and gender**

IDS quality and quantity vary as children develop. The mean f0 seems to increase from birth, peak at approximately 4 to 6 months, and decrease slowly until the age of two years or older [47,48]. Acoustic exaggeration is also smaller in child-directed speech (CDS) than in IDS [49]. Prosodic contours vary with infants’ age [50], with “comforting” prevalent between 0 and 3 months and then decreasing with age, “expressing affection” and “approval” peaking at 6 months and being least evident at 9 months, and “directive” utterances, which are rare at birth, peaking at 9 months of age [47]. This is consistent with a change in pragmatic function between 3 and 6 months of age, as parental speech becomes less affective and more informative [3]. Variations in the mean length of utterances (MLU) are more controversial, and Soderstrom emphasized that some properties, such as linguistic simplifications, could be beneficial at one age but problematic at another age. In fact, two small sample studies suggest that mothers adjust their IDS as a function of their children's language ability. Around the two-word utterance period, an adult-like conversational style with frequent overlaps emerges in Japanese IDS [51], which has a mean f0 that reaches approximately the same value as that of ADS [52]. Mothers may continue to adjust their speech syntax to their children’s age and to the child’s feedback as children grow older [53], but more longitudinal studies investigating the evolution of the linguistic aspects of IDS are needed.

Infants’ gender may also modify IDS characteristics. When using IDS with their 0- to 12-month-old infants, Australian mothers used higher f0 and f0 ranges and more rising utterances for girls than boys, whereas Thai mothers used a more subdued mean f0 and more falling utterances for girls than boys [54]. Given that the gender of the infant is not neutral in interactional processes (see, for example 55), its impact on motherese should be further explored in motherese studies.

**3.3. According to infant vocalizations, ability and reactivity**

Gleason suggested that children’s feedback helps shape the language behavior of those who speak to them [56]. Indeed, Fernald, in a comparison of IDS, simulated IDS (to an absent baby) and ADS, showed that an infant’s presence facilitates IDS production. In simulated IDS, the mean f0 did not rise significantly compared with that of ADS, and other features, though they differed significantly from ADS, were intermediately between those of IDS and ADS [9]. In fact, IDS is dynamically affected by infants’ feedback. For example, IDS is reduced when the contingency of an infant’s responses is disturbed by decoupling TV sequences of the mother-infant interaction [57]. Furthermore, mothers produce higher IDS pitch when, through an experimental manipulation, IDS high pitch seems to elicit infants’ engagement, compared to another manipulation in which low pitch seems to strengthen infant’s engagement [58]. Mothers may also match their pitch to infants’ vocalizations. In the first 3 months, IDS and infants’ vocalizations are correlated in pitch, and even melody types are correlated in some mother-infant pairs [59] with tonal synchrony [60]. This correlation may be due to the parents, given that, in a longitudinal case study, parents consistently adjusted their vocal patterns to their 3- to 17-month-old infants’ vocal patterns, whereas infants did not adjust their vocal patterns to their parents’ vocal patterns [61]. 

In addition, mothers adapt their IDS to infants’ abilities and needs. A number of studies have shown that mothers strengthen their IDS according to the perceived lack of communicative abilities of their child. Although full-term infants more often followed their mothers’ utterances with a vocalization than preterm infants did, mothers of premature babies more often followed their infants' vocalizations with an utterance directed at the infants than did mothers of full-term babies [62]. A mother of two 3-month-old fraternal twins accommodated her IDS by using a higher mean f0 and rising intonation contours when she spoke to the infant whose vocal responses were less frequent [63]. Similarly, playing with a Jack-in-the-box, mothers exclaimed in surprise with a higher pitch when their children did not show a surprise facial expression. Infants’ expressions were a stronger predictor of maternal vocal pitch than their ages [64]. Mothers interacting with an unfamiliar deaf 5-year-old child used more visual communicative devices, touches, simpler speech, and frequent initiations than when communicating with an unfamiliar hearing 4.5-year-old child. Although each initiation toward the deaf child was less successful than the previous one, interactions occurred as frequently as with the hearing child [65]. Finally, parents of children with Down syndrome (which is a visible disability) spoke with a significantly higher f0 mean and variance than did parents of children with other types of mental retardation [66].

Mothers tailor their communication to their infants' levels of lexical-mapping development. When teaching their infants target words for distinct objects, mothers used target words more often than non-target words in synchrony with the object’s motion and touch. This mothers’ use of synchrony decreased with infants' decreasing reliance on synchrony as they aged [67]. Similarly, IDS’s semantic content shows strong relationships with changes in children's language development from zero to one-word utterances [68], and a clear signal of non-comprehension from children results in shorter utterances [69]. In the IDS directed toward their profoundly deaf infants with cochlear implants, mothers tailored pre-boundary vowel lengthening to their infants' hearing experience (i.e., linguistic needs) rather than to their chronological age, yet they all exaggerated the prosodic characteristics of IDS (i.e., affective needs) regardless of their infants' hearing status [70,71]. Thus, we conclude that IDS largely depends on the child given that it increases with infants’ presence and engagement, is influenced by infants’ actual preferences and vocalizations and depends on mothers’ perceptions of their infants’ overall abilities and needs.

**3.4. Do parental individual differences modify motherese quality?**

Whether a mother had siblings could explain some individual variability in IDS, given that women who grew up with siblings were more likely to show prosodic modifications when reading picture books to a young child than those who did not have siblings [72]. Social class and socio-economic status (as measured by income and education) impact mothers' CDS [73-75], and this impact is mediated by parental knowledge of child development [75]. However, main effects of communicative setting (e.g., mealtime, dressing, book reading, or toy play) and the amount of time that mothers spend interacting with their children may be important influences [74]. 

Neural and physiological factors may be relevant to the parenting of young children and to IDS production. When listening to IDS, mothers of preverbal infants (unlike mothers of older children) showed enhanced activation in the auditory dorsal pathway of the language areas in functional MRIs. Higher cortical activation was also found in speech-related motor areas among extroverted mothers [76]. Additionally, in the first 6 months, the maternal oxytocin level is related to the amount of affectionate parenting behavior shown, including "motherese" vocalizations, the expression of positive affect, and affectionate touch [77].

Finally, maternal pathology may affect IDS. The influence of maternal depression on IDS has been the main focus of previous studies. Results show that depressed mothers fail to modify their behavior according to the behavior of their 3- to 4-month-old infants, are slower to respond to their infants’ vocalizations, and are less likely to produce motherese [78]. Depressed mothers also speak less frequently with fewer affective and informative features with their 6- and 10-month-old infants, and the affective salience of their IDS fails to decrease over time [79]. Moreover, depressed mothers show smaller IDS f0 variance except when taking antidepressant medication and being in partial remission [80]. Mothers with schizophrenia also show less frequent use of IDS compared to other mothers with postnatal hospitalizations [81].

Thus, in addition to factors associated with the infants, various maternal factors (i.e., familial, socio-economic, physiological, and pathologic) can modulate IDS production.

### 4: Motherese effects on the infant

 As hypothesized, IDS may function developmentally to communicate affect, regulate infants’ arousal and attention, and facilitate speech perception and language comprehension [16,82].

**4.1. Communication of affect and physiological effects**

Though communication of affect is crucial with regard to communicating with very young infants without linguistic knowledge, few studies have addressed it. Despite the lack of available studies, IDS may convey mothers’ affect and influence infants’ emotions. As reported previously, prosodic patterns are more informative in IDS, and the variations in prosodic contours provide infants with reliable cues for determining their mothers’ affect and intentions. Indeed, when hearing an unfamiliar language in IDS, 5-month-olds smile more often to approvals and display negative affect in response to prohibitions, and these responses were not evident in ADS [83]. Similarly, IDS approval contours elevate infants’ looking, whereas disapproval contours inhibit infants’ looking [84]. Also 14-18 months old infants use prosody to understand intentions [85]. At a psycho-physiological level, a deceleration in heart rate was observed in 9-month-old infants listening to IDS, and EEG power, specifically in the frontal region, was linearly related to the affective intensity of IDS [86]. Finally, one study [87] reported an astonishing physiological correlation: 3- to 4-month-old infants (N=52) grew more rapidly when their primary caregivers spoke high quality/quantity IDS. This could be influenced by other intermediate physiological factors, but this work needs to be replicated.

**4.2. Facilitation of social interactions through infants’ preference for IDS**

Infants prefer to listen to IDS when compared to ADS [88], and they show greater affective responsiveness to IDS than ADS [89]. This finding is also evident for deaf infants seeing infant-directed signing [44] and even for severely handicapped older hearing children [89]. Moreover, infants remember and look longer at individuals who have addressed them with IDS [90]. Finally, this greater responsiveness makes them more attractive to naïve adults, which helps maintain positive adult-infant interactions [91].

Infants’ preferences follow a developmental course, on that they are present from birth and may not depend on any specific postnatal experience (though prenatal auditory experience with speech may play a role). One-month-old and even newborn infants prefer the IDS from an unfamiliar woman to the ADS from the same person [92-94]. While neonates sleep, the frontal cerebral blood flow increases more with IDS than with ADS, which suggests that IDS alerts neonates’ brains to attend to utterances even during sleep [95]. IDS preferences change with development, in that the preference for IDS decreases by the end of the first year [96,97]. Thereafter, infants may be more inconsistent, in that one study found a preference for IDS [96] but another did not [97]. Thus, more studies are needed to understand the precise course of infants’ preferences for IDS after 9 months of age. With regard to the speech of their own mothers, only 4-month-old infants (and not 1-month-olds) prefer IDS to ADS [98], and newborns prefer their mothers' normal speech to IDS [99]. With regard to the quality of IDS, infants’ preferences also follow a developmental course. Four-month-olds prefer slow IDS with high affect, whereas 7-month-olds prefer normal to slow IDS regardless of its affective level [100]. The developmental course of infants' preferences is consistent with the type of affective intent used by mothers at each age [47]. The terminal falling contour of IDS (e.g., a comforting utterance) may serve to elicit a higher rate of vocal responses in 3-month-old infants [101]. Infants’ preferences shift between 3 and 6 months from comforting to approving, and between 6 and 9 months from approving to directing [102]. Rising, falling, and bell-shaped IDS contours arouse 4- to 8-month-olds’ attention [103]. However, 6-month-olds, but not 4-month-olds, are able to categorize IDS utterances into approving or comforting [104]. Finally, adults prefer ADS (i.e., in content and prosody) to IDS [105]. 

What are the acoustic determinants of infants’ preference for IDS? When lexical content is eliminated, young infants show an auditory preference for the f0 patterns of IDS, but not for the amplitude (correlated to loudness) or duration patterns (rhythm) of IDS [88,106,107]. This pattern is consistent with the finding that infants prefer higher pitched singing [108]. However, deaf infants also show greater attention and affective responsiveness to infant-directed signing than to adult-directed signing [44]. Although an auditory stimulus with IDS characteristics was more easily detected in noise than one that resembled ADS characteristics [109] and mothers accentuate some IDS characteristics in a noisy context [97], infants’ preference is independent of background noise [97]. Actually, IDS preference relies on a more general preference for positive affect in speech. When affect is held constant, 6-month-olds do not prefer IDS. They even prefer ADS if it contains more positive affect than IDS. Having a higher and more variable pitch is neither necessary nor sufficient for determining infants' preferences, although f0 characteristics may modulate affect-based preferences [110]. This result may be linked with the finding that IDS’s prosody is driven by the widespread expression of emotion toward infants compared with the more inhibited manner of adult interactions [22]. However, though this issue may be very fruitful for future study, as was evident in the previous section, there is currently a lack of studies addressing the affective and emotional effects of motherese (for example, the immediate effects on infants’ expressions, variations according to infants’ age, later effects on infants’ attachment, and so on, as for mother-infant synchrony, the immediate and later effects of which are now well documented). In contrast, many studies simply address the more behavioral concept of “infants’ preference” for motherese. Finally, preferences depend on linguistic needs. Six-month-olds, for example, prefer IDS that is directed at older infants when the frequency of repeated utterances is greater, thus matching the IDS directed at younger infants [111]. This preference for repetitiveness may explain why 6-month-olds prefer audiovisual episodes of their mothers singing rather than speaking IDS [112,113]. 

In summary, the preference for IDS, which is characterized by better attention, gaze and responsiveness from infants, is less prevalent for the infant’s own mother, and is generally related to the affective intensity of the voices. Moreover, this preference is modulated by the age of the infant, which is most likely due to infants’ affective and cognitive abilities and needs. 

**4.3. Arousing infants’ attention and learning**

IDS has arousing properties and facilitates associative learning. In contrast to ADS, IDS elicits an increase in infants’ looking time between the first and second presentations. Similarly, when alternating ADS and IDS, infants’ responses to ADS are stronger if preceded by IDS, whereas their responses to IDS are weaker if preceded by ADS [114]. In a conditioned-attention paradigm with IDS or ADS as the signal for a face, only IDS elicited a significant positive summation, and only when presented with a smiling or a sad face (not a fearful or an angry one) [115]. IDS may in fact serve as an ostensive cue, alerting a child to the referential communication directed at him or her. Eye-tracking techniques revealed that 6-month-olds followed an adult's gaze (which is a potential communicative-referential signal) toward an object (i.e., joint attention) only when it was preceded by ostensive cues, such as IDS or a direct gaze [116]. Likewise, the prosodic pattern of motherese (which is similar to other cues such as eye contact, saying the infant’s name and contingent reactivity) triggered 14-month-olds to attend to others' emotional expressions that were directed toward objects [117]. Thus, IDS may help infants learn about objects from others and, more specifically, about others’ feelings toward these objects, which may pave the way for developing a theory of mind and intersubjectivity.

Yet, experience-dependent processes also influence the effects of IDS. Kaplan conducted several studies using the same conditioned-attention paradigm with face reinforcers to assess how parental depression affected infants’ learning. We know that depression reduces IDS quantity [78] and quality [80], which may explain why infants of depressed mothers do not learn from their mother’s IDS yet still show strong associative learning in response to IDS produced by an unfamiliar, non-depressed mother [118,119]. However, this learning was poorer when maternal depression lasted longer (e.g., with 1-year-old children of mothers with perinatal onset) [120]. Nevertheless, infants of chronically depressed mothers acquired associations from the IDS of non-depressed fathers [121]. Paternal involvement may also affect infants’ responsiveness to male IDS. In contrast with infants of unmarried mothers, infants of married mothers learned in response to male IDS, especially if their mothers were depressed [122]. However, as expected, infants of depressed fathers showed poorer learning from their fathers' IDS [123]. Finally, current mother-infant interactions influence infants’ learning from their mothers’ IDS. In fact, f0 modulations, though smaller in depressed mothers’ IDS, did not predict infants’ learning, whereas maternal sensitivity did, even when accounting for maternal depression [124]. In summary, IDS learning facilitation is affected by past and current experiences (such as long durations of time with a depressed mother, having an involved father, and having a sensitive mother).

**4.4. Facilitation of language acquisition**

4.4.1. Does IDS’s prosody aid in language acquisition, and, if so, how?

The supra-segmental characteristics of IDS (i.e., f0 amplitude and duration) can facilitate syllable discrimination [125,126]. When given vowel tokens that were drawn from either English or Japanese IDS, an algorithm successfully discovered the language-specific vowel categories, thereby reinforcing the theory that native language speech categories are acquired through distributional learning [127]. Trainor observed that, although the exaggerated pitch contours of IDS aid in the acquisition of vowel categories, the high pitch of IDS might impair infants' ability to discriminate vowels (thereby serving a different function, such as attracting infants' attention or aiding in their emotional communication) [128]. Nevertheless, IDS’s prosody facilitates syllabic discrimination and vowel categorization in the first 3 months.

IDS’s prosody may also help pre-linguistic infants segment speech into clausal units that have grammatical rules, and the pitch peaks of IDS, especially at the ends of utterances, may assist in word segmentation and recognition, which facilitates speech processing. Indeed, 7- to 10-month-olds prefer to listen to speech samples that are segmented at clause boundaries than to samples with pauses inserted at within-clause locations [129], but this was only for IDS samples, not for ADS samples [130]. Infants can distinguish words from syllable sequences that span word boundaries after exposure to nonsense sentences spoken with IDS’s prosody, but not with ADS’s prosody [131]. Moreover, mothers of 20-month-old late-talkers marked fewer nouns with a pitch peak and used more flat pitch contours than mothers of typical children [132]. In a review of previous research, Morgan suggested that prosody is an important contributor to early language understanding and assists infants in developing the root processes of parsing [133]. 

Stress information shapes how statistics are calculated from the speech input and is encoded in the representations of the parsed speech sequences. For example, to parse sequences from an artificial language, 7- and 9-month-olds adopted a stress-initial syllable strategy and appeared to encode the stress information as part of their proto-lexical representations [134]. In fluent speech, 7.5-month-olds prefer to listen to words produced with emphatic stress, although recognition was most enhanced when the degree of emphatic stress was identical during familiarization and recognition tasks [135]. Does word learning with IDS’s prosody impair word recognition in ADS? The high affective variation in IDS appears to help preverbal infants recognize repeated encounters with words, which creates both generalizable representations and phonologically precise memories for the words. Conversely, low affective variability appears to degrade word recognition in both aspects, thereby compromising infants' ability to generalize across different affective forms of a word and detect similar sounding items [136]. Automatic isolated-word speech recognizers trained on IDS did not always generate better recognition performances, but, for mismatched data, their relative loss in performance was less severe than that of recognizers trained on ADS, which may be due to the larger class overlaps in IDS [137]. Additionally, 7- to 8-month-old infants were successful on word recognition tasks when words were introduced in IDS and not successful for those introduced in ADS, regardless of the register of recognition stimuli [138]. Furthermore, IDS may be more easily detected than ADS in noisy environments [109]. Finally, clarity may vary with the age of the listener. Having a slow speaking rate and vowel hyper-articulation improved 19-month-olds’ ability to recognize words, but having a wide pitch range did not [139]. For adult listeners, words that were isolated from parents’ speech to their 2- to 3-year-olds were less intelligible than words produced in ADS [140]. 

Thus, IDS’s prosody facilitates vowel categorization, syllabic discrimination, speech segmentation in words and grammatical units, and word recognition. Moreover, IDS’s prosody may serve as an attentional spotlight that increases brain activity to potentially meaningful words [141]. Indeed, event-related potentials increased more for IDS than ADS (only in response to familiar words for 6-month-olds and to unfamiliar words for 13-month-olds). 

4.4.2. Do the linguistic properties of IDS aid in language acquisition, and, if so, how?

In response to both Chomsky’s view that motherese is a form of degenerate speech and the resulting theoretical impetus toward nativist explanations of language acquisition, several researchers have sought for evidence that language input to children is highly structured and possibly highly informative for the learner. There has been a lively debate between the proponents of motherese as a useful tool for language acquisition and those who contend that it does not aid language acquisition. First, Newport [142] claimed that motherese is not a syntax-teaching language, given that it may be an effect rather than a cause of learning language. Newport and colleagues found few correlations between the syntax evident in caregivers’ speech and language development. Responding to Furrow [143], one study with two groups of age-matched children (18- to 21-month-olds and 24- to 27-month-olds) also found few effects of the syntax of mothers’ IDS on children's language growth, with most effects restricted to a very young age group, which suggested that the complexity of maternal speech is positively correlated with child language growth in this age range [144]. Scarborough [145] also found that maternal speech type did not influence language development.

However, other studies that considered children's level of language at the time of maternal speech assessment found a relationship between maternal IDS’s semantic and syntactic categories and children’s language development. Several characteristics (e.g., MLU and pronoun use) of mothers’ IDS with their 18-month-olds predicted the children’s subsequent (27-month-old) speech, specifically, the mothers' choice of simple constructions facilitated language growth [143,146]. Rowe, in a study controlling for toddlers’ previous vocabulary abilities, found that CDS at 30 months of age predicted children’s vocabulary ability one year later [75]. As early as 13 months of age, pre-existing differences were found between mothers of earlier and later talkers. When individual differences in style of language acquisition (i.e., expressive versus non-expressive styles) were examined, several associations emerged for the “non-expressive” group between the IDS type at 13 months of age and the mean length of utterance at 20 months of age [147].

Which linguistic characteristics of motherese may aid in language acquisition? First, the statistically prominent structural properties of CDS that may facilitate language acquisition are present in realistic CDS corpora [148]. In particular, the partial overlap of successive utterances, which is well known in CDS, enhances adults’ acquisition of syntax in an artificial language [149]. CDS contains isolated words and short, frequently used sentence frames. A familiar sentence context may aid in word acquisition given that 18-month-olds are slower to interpret target words (i.e., familiar object names) in isolation than when these words are preceded by a familiar carrier phrase [150]. The tendency in IDS to put target words in sentence-final positions may help infants segment the linguistic stream. When hearing IDS in Chinese, English-speaking adults learned the target words only when the words were placed in the final position, and this was not when they were placed in a medial position [151]. Finally, the use of diminutives (a pervasive feature of CDS that is evident in many languages) facilitates word segmentation in adults hearing an unfamiliar language [152,153], and enhances gender categorization [154] and gender agreement even in languages that uses few diminutives [155].

In summary, results support the idea that prosodic and linguistic aspects of IDS play an important role in language acquisition. One possibility is that prosodic components play a major part in the very early stages of language acquisition and linguistic aspects play an increasingly important part later in development when children gain some verbal abilities. 

## Discussion

### 1: Summary

 Our review has some limitations. Some studies may have not been identified because not recorded in our 2 databases. Some studies without significant results may have not been reported (risk of a publication bias). And some results of included studies may be considered with caution because they don’t have been replicated or they sometimes derive from a little sample of participants. Some highlights emerge from this review, however. IDS transcends specific languages. Mothers, fathers, grandmothers and other caregivers all modify their speech when addressing infants, and infants demonstrate a preference for IDS. Nonetheless, various factors related either to the caregiver or to the infant influence the quality of the IDS. If present from birth, IDS, like an infant’s preference for IDS, follows a developmental course that can be influenced by the infant’s experience (see Kaplan’s work). IDS consists of linguistic and supra-linguistic modifications. The linguistic modifications include shorter utterances, vocabulary and syntactic simplifications, and the use of diminutives and repetition, all of which are designed to facilitate comprehension and aid in language acquisition. Prosodic modifications may serve more ubiquitous functions. Using a higher pitch matches infants’ preferences, and, using a wider f0 range may facilitate infants’ arousal and learning. Prosodic contours convey caregivers’ affect and intentions, and some of these contours stimulate infants’ responsiveness. Finally, exaggerated pitch contours and phonetic modifications facilitate vowel discrimination, phonetic categorization, speech segmentation, word recognition and syntax acquisition. 

### 2: Positioning IDS within a More Global Communication Phenomenon

 We observed that mothers adjust their IDS to their infants’ abilities. From a broader communications perspective, IDS may be part of a more general phenomenon of adaptation to a partner during communication. First, other cases of speech adjustment to the listener exist. Adults simplify their vocabulary choices when speaking with children who are up to 12 years of age [156]. In speech directed at elderly adults, CDS (which clarifies instructions by giving them in an attention-getting manner) is often used and may improve elderly adults’ performance and arousal in difficult tasks [157]. Even in normal ADS, new words are highlighted with prosodic cues. In both IDS and ADS, repeated words are shorter, quieter, lower pitched, and less variable in pitch than words the first time they are mentioned, and they are placed in less prominent positions relative to new words in the same utterance [5]. Even in master–dog dyads, the structural properties of “doggerel” (PDS) are strikingly similar to the structural properties of motherese except in functional and social areas [158]. Second, speakers other than human mothers and caregivers adjust their speech to infants. Four-year-old children modify some of their prosodic characteristics when speaking to infants, in that they speak more slowly, tend to lower their f0, and change their amplitude variability [159]. The linguistic content of educational children's programs also generally follows the linguistic constraints and adjustments that are evident in adults' CDS [160]. The use of IDS by humans has been compared with the “caregiver call” (which is almost exclusively infant-directed) in squirrel monkeys, of which the variability of several acoustic features, most notably pitch range and contour, is associated with particular contexts of infant care, such as nursing or retrieval [161]. Similarly, tamarins are calmed by music with the “acoustical characteristics of tamarin affiliation vocalizations” [162]. In a comparison of the mother-infant gestural and vocal interactions of chimpanzees and humans, Falk [163], suggested that pre-linguistic vocal substrates for motherese evolved as females gave birth to relatively undeveloped neonates and adopted new strategies that entailed maternal quieting, reassuring, and controlling of the behaviors of physically removed infants (who were unable to cling to their mothers' bodies). The characteristic vocal melodies of human mothers' speech to infants might be biologically relevant signals that have been shaped by natural selection [164], a finding that is integrated in a more general human and non-human communication field.

### 3: Integrating IDS into the Nature of Mother-Infant Interactions

 IDS implies emotion sharing, mother-infant adjustment, synchrony and multimodal communication. Indeed, IDS is part of a multimodal, synchronous communication style used with infants to sustain interactions and highlight messages. Mothers support their vocal communication with other modalities (e.g., gestural, tactile, and visual). At a gestural level (“gesturese”), mothers of 16- and 20-month-old infants employ mainly concrete deictic gestures (e.g., pointing) that are redundant with the message being conveyed in speech to disambiguate and emphasize the verbal utterance. Moreover, children's verbal and gestural productions and vocabulary size may be correlated with maternal gesture production [165,166]. Mothers’ demonstrations of the properties of novel objects to infants are higher in interactiveness, enthusiasm, proximity to the partner, range of motion, repetitiveness and simplicity, thereby indicating that mothers modify their infant-directed actions in ways that likely maintain infants' attention and highlight the structure and meaning of an action [167]. Moreover, mothers’ singing and synchronous behaviors with the beat (“songese”) segment the temporal structure of the interaction, such that 3- to 8-month-old infants are sensitive to their mothers’ emphasis by producing more synchronous behaviors on some beats than on others. The multimodal sensory information provided by mothers shares the characteristics of “motherese” and may ensure effective learning in infants [168]. Mothers also use contingency and synchrony (both intrapersonal and interpersonal) to reinforce dialogues and exchanges. By highlighting focal words using the nonlinguistic contextual information that is available to the listener and by producing frequent repetitions and formulaic utterances, IDS may be a form of “hyper-speech” that facilities comprehension by modifying the phonetic properties of the individual words and providing contextual support on perceptual levels that are accessible to infants even in the earliest stages of language learning [169]. Pragmatic dimensions of IDS may provide contingent support that assists in language comprehension and acquisition. In a case study, both parents used approximately equal amounts of language with their infants, but the functions of the mother’s speech differed importantly from those of the father’s speech with regard to providing more interactive negotiations, which could be crucial to language development [170]. Thus, IDS appears to be a part of a maternal interactive style that supports the affective and verbal communication systems of the developing infant.

 IDS should be regarded as an emotional form of speech. Several studies highlight the impact of emotion on both motherese production and its effects, particularly with regard to prosodic characteristics that are conditioned by vocal emotions [22]. In general, acoustic analyses of f0 are positively associated with subjective judgments of emotion [171]. Thus, prosody (which is linked with f0 values and contours) reveals affective quantity and quality. The literature on infants' perception of facial and vocal expressions indicates that infants’ recognition of affective expressions relies first on multimodally presented information, then on recognition of vocal expressions and finally on facial expressions [172]. Moreover, IDS’s affective value determines infants’ preferences [110]. Therefore, mothers’ affective pathologies, which include maternal depression, alter motherese and impair infants’ conditioned learning with IDS. Could IDS, music and emotion be linked before birth through prenatal associations between a mother's changing emotional state, concomitant changes in hormone levels in the placental blood and prenatally audible sounds? These links may be responsible for infants’ sensitivity to motherese and music [173]. 

Finally, IDS highlights mother-infant adjustments during interactions. Mothers adjust their IDS to infants’ age, cognitive abilities and linguistic level. Therefore, IDS may arouse infants’ attention by signaling speech that is specifically addressed to them, with content and form that are adapted for them. Mothers also adapt their IDS to infants’ reactivity and preferences. Mothers’ continuous adjustments to their infants result in the facilitation of exchanges and interactions, with positive consequences for sharing emotions and for learning and language acquisition. Thus, maternal sensitivity predicts infants’ learning better than f0 ranges do [124]. Infants’ reactivity is also important given that their presence increases motherese [9], and infants’ positive, contingent feedback makes them more attractive [91], which in turn increases the quality of the motherese [57,58]. Mother-infant contingency and synchrony are crucial for IDS production and prolongation.

**Figure 2:**
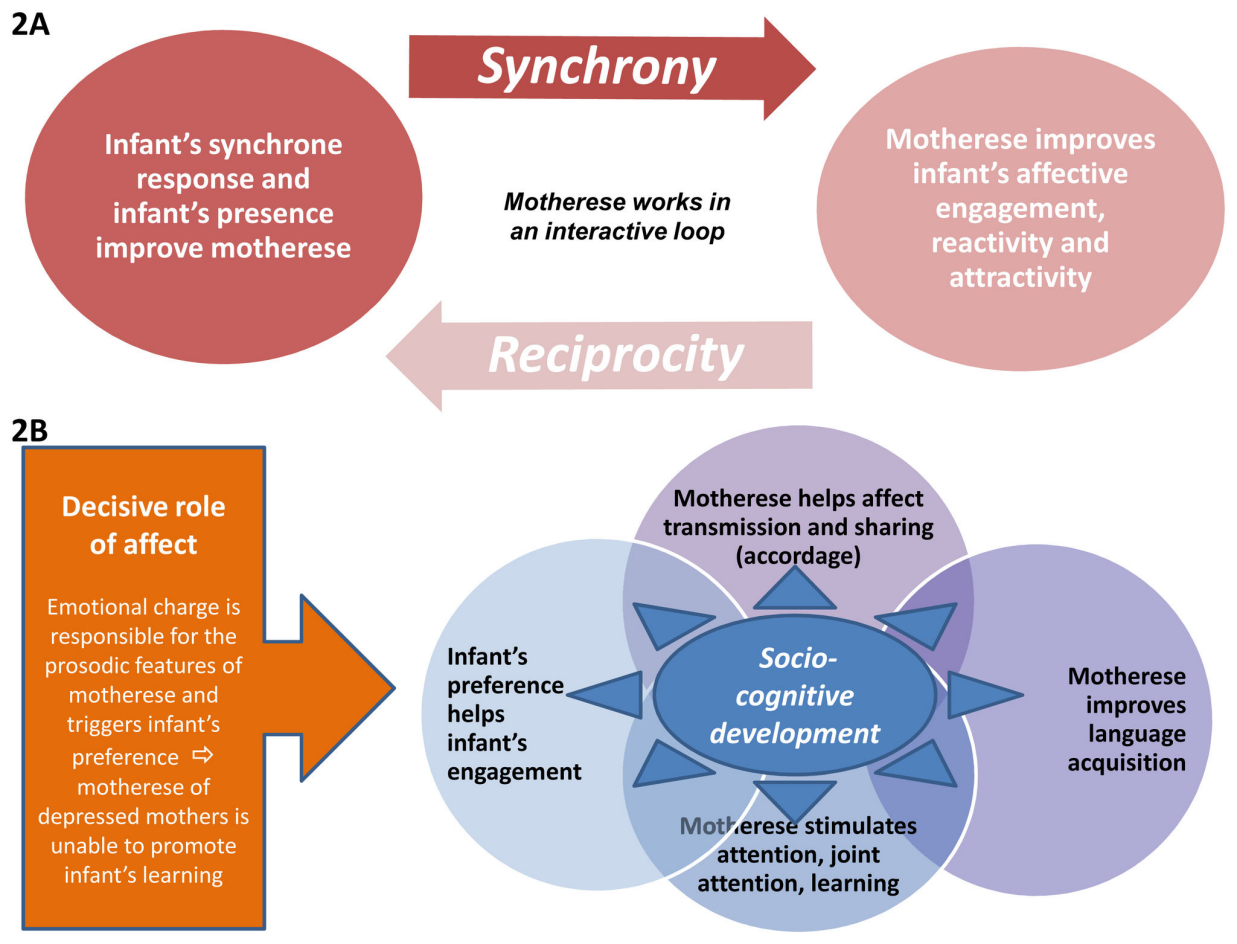
Summary of the motherese interactive loop (a) and its socio-cognitive implications (2B). 1A: The motherese interactive loop implies that motherese is both a vector and a reflection of mother-infant interaction. 2B: Motherese affects intersubjective construction and learning. Its implications for infants’ early socio-cognitive development are evident in affect transmission and sharing, and in infants’ preferences, engagement, attention, learning and language acquisition.

In Figure 2, we summarize the main points that were previously discussed. We suggest that motherese mediates and reflects an interactive loop between the infant and the caregiver, such that each person’s response may increase the initial stimulation of the other partner. At the behavioral level, this interactive loop is underpinned by the emotional charge of the affective level and affects, at the cognitive level, attention, learning and the construction of intersubjective tools, such as joint attention and communicative skills. Direct evidence of this intertwinement of cognitive and interactive levels is offered by Kuhl’s finding that infants’ learning of the phonetic properties of a language requires interactions with a live linguistic partner [174], as audiovisual input is insufficient for this. Regarding this impact of social interaction on natural speech and language learning, Kuhl wondered whether the underlying mechanism could be the increased motivation, the enriched information that social settings provide, or a combination of both factors [175]. Given that autistic children and children raised in social deprivation do not develop a normal language, Kuhl suggested that the social brain “gates” language acquisition. As an outcome of our review, we suggest that the co-construction that emerges from the reciprocal infant-maternal adaptation and reinforcement via the interactive loop could be crucial to the development of infants’ cognitive and verbal abilities, which would be consistent with humans’ fundamental social nature. 

## Conclusion

 Some authors held the perspective that, beyond language acquisition, IDS significantly influences infants’ cognitive and emotional development (e.g., [4,176]). Our systematic review supports this view. More studies are needed to understand how IDS impacts affective factors in infants and how this is linked with infants’ cognitive development, however. An interesting approach may be to investigate how this process is altered by infants’ communicative difficulties, such as early signs of autism spectrum disorder, and how these alterations may affect infants’ development [177].

## Supporting Information

Annex S1 **Rejected papers and reasons for their exclusion.**
(DOCX)

Checklist S1 **PRISMA Checklist.**
(DOCX)
