# Supplementary material for: Motherese in Interaction: At the Cross-Road of Emotion and Cognition? (A Systematic Review)
Source: PLoS One. 2013 Oct 18;8(10):e78103. doi: 10.1371/journal.pone.0078103 (PMC3800080; doi:10.1371/journal.pone.0078103)
Supplement: Annex S1 — Rejected papers and reasons for their exclusion. (DOCX) [file pone.0078103.s001.docx]

# Annex S1: Rejected papers and reasons for their exclusion.

Literature reviews or discussion [1,55,56,82,133,146,163,169,172,173,176]

Studies dealing with other than adult-infant interactions [156,157,159];

Studies dealing with vocalizations to or from animals [161,162];

Studies dealing with motion or gestures [165-167];

Studies dealing with singing [113,168];

Study on functional aspects of language input, in a language acquisition perpective [170].

Paper reviewing infants’ early perceptual processing of speech **[178];**

Study addressing infants’ discrimination of adult-directed speech (ADS) dysfluency [3];

Articles studying global caregiver-child interactions [112,**179-182**];

Study investigating not the form but the thematic properties of the speech mothers address to infants [**183**];

Studies using IDS as a stimulus or a reference while addressing other questions [171,**184-193**];

Studies addressing, across different languages, the influence of ambient language or IDS on newborn cries [**194**] or infant babbling [36,**195**];

Study focusing on the speech of children with autism spectrum disorders [**196**].

Papers using IDS samples or IDS properties to better understand language acquisition (word segmentation, acquisition of syntax, grammatical categories, phonetic categories, and similar topics) [46,**197-201**].

List of excluded studies and not cited in the reference list of the paper (in bold):

178. Cooper RP, Aslin RN (1989) The language environment of the young infant: Implications for early perceptual development. Canadian Journal of Psychology/Revue canadienne de psychologie 43: 247-265.

179. Rivero M (2010) Maternal expression of communicative intentions and pragmatic fine tuning in early infancy. Infant Behavior and Development 33: 373-386.

180. Jones R (2003) Listen and learn. Nature Reviews Neuroscience 4.

181. Eckerman CO, Oehler JM, Hannan TE, Molitor A (1995) The development prior to term age of very prematurely born newborns' responsiveness in En Face exchanges. Infant Behavior & Development 18: 283-297.

182. Eckerman CO, Oehler JM, Medvin MB, Hannan TE (1994) Premature newborns as social partners before term age. Infant Behavior & Development 17: 55-70.

183. Rabain-Jamin J, Sabeau-Jouannet E (1997) Maternal speech to 4-month-old infants in two cultures: Wolof and French. International Journal of Behavioral Development 20: 425-451.

184. Zentner M, Eerola T (2010) Rhythmic engagement with music in infancy. Proc Natl Acad Sci U S A 107: 5768-5773.

185. Shinozaki T, Ostendorf M, Atlas L (2009) Characteristics of speaking style and implications for speech recognition. J Acoust Soc Am 126: 1500-1510.

186. Pena M, Maki A, Kovacic D, Dehaene-Lambertz G, Koizumi H, et al. (2003) Sounds and silence: an optical topography study of language recognition at birth. Proc Natl Acad Sci U S A 100: 11702-11705.

187. Waxman SR (1999) Specifying the scope of 13-month-olds' expectations for novel words. Cognition 70: B35-50.

188. Kuhl PK, Coffey-Corina S, Padden D, Dawson G (2005) Links between social and linguistic processing of speech in preschool children with autism: behavioral and electrophysiological measures. Dev Sci 8: F1-F12.

189. Wagner L, Greene-Havas M, Gillespie R (2010) Development in children's comprehension of linguistic register. Child Development 81: 1678-1686.

190. Brand RJ, Tapscott S (2007) Acoustic packaging of action sequences by infants. Infancy 11: 321-332.

191. McCartney JS, Panneton R (2005) Four-Month-Olds' Discrimination of Voice Changes in Multimodal Displays as a Function of Discrimination Protocol. Infancy 7: 163-182.

192. Ward CD, Cooper RP (1999) A lack of evidence in 4-month-old human infants for paternal voice preference–59. Developmental Psychobiology 35: 49-59.

193. Kaplan PS, Zarlengo-Strouse P, Kirk LS, Angel CL (1997) Selective and nonselective associations between speech segments and faces in human infants. Developmental Psychology 33: 990-999.

194. Mampe B, Friederici AD, Christophe A, Wermke K (2009) Newborns' cry melody is shaped by their native language. Curr Biol 19: 1994-1997.

195. Chen LM, Kent RD (2010) Segmental production in Mandarin-learning infants. J Child Lang 37: 341-371.

196. Sharda M, Subhadra TP, Sahay S, Nagaraja C, Singh L, et al. (2010) Sounds of melody--pitch patterns of speech in autism. Neurosci Lett 478: 42-45.

197. Fisher C, Tokura H (1996) Acoustic cues to grammatical structure in infant-directed speech: cross-linguistic evidence. Child Dev 67: 3192-3218.

198. Shi R, Morgan JL, Allopenna P (1998) Phonological and acoustic bases for earliest grammatical category assignment: a cross-linguistic perspective. J Child Lang 25: 169-201.

199. Lee S, Davis BL, MacNeilage P (2007) 'Frame dominance' and the serial organization of babbling, and first words in Korean-Learning infants. Phonetica 64: 217-236.

200. Lebedeva GC, Kuhl PK (2010) Sing that tune: infants' perception of melody and lyrics and the facilitation of phonetic recognition in songs. Infant Behav Dev 33: 419-430.

201. Pelucchi B, Hay JF, Saffran JR (2009) Statistical learning in a natural language by 8-month-old infants. Child Development 80: 674-685.
